# In-depth characterization of NK cell markers from CML patients who discontinued tyrosine kinase inhibitor therapy

**DOI:** 10.3389/fimmu.2023.1241600

**Published:** 2023-09-25

**Authors:** María Belén Sanchez, Bianca Vasconcelos Cordoba, Carolina Pavlovsky, Beatriz Moiraghi, Ana Varela, Rosario Custidiano, Isolda Fernandez, María Josefina Freitas, María Verónica Ventriglia, Georgina Bendek, Romina Mariano, María José Mela Osorio, Miguel Arturo Pavlovsky, Ana García de Labanca, Cecilia Foncuberta, Isabel Giere, Masiel Vera, Mariana Juni, José Mordoh, Julio Cesar Sanchez Avalos, Estrella Mariel Levy, Michele Bianchini

**Affiliations:** ^1^ Centro de Investigaciones Oncológicas, Fundación Cáncer (CIO-FUCA), Ciudad Autónoma de Buenos Aires, Buenos Aires, Argentina; ^2^ Hematology Department, Fundación para combatir la leucemia (FUNDALEU), Buenos Aires, Argentina; ^3^ Hematology Department, Hospital José María Ramos Mejía, Buenos Aires, Argentina; ^4^ Hematology Department, Instituto Alexander Fleming, Buenos Aires, Argentina; ^5^ Hematology Department, Hospital Posadas, Buenos Aires, Argentina; ^6^ Hematology Department, Hospital Italiano, Buenos Aires, Argentina; ^7^ Hematology Department, Hospital San Martín, Paraná, Entre Ríos, Argentina; ^8^ Hematology Department, Hospital Italiano de Mendoza, Mendoza, Argentina

**Keywords:** chronic myeloid leukemia, treatment free remission, NK cells, degranulation, immunophenotype

## Abstract

**Introduction:**

Treatment-free remission (TFR) in patients with chronic myeloid leukemia in chronic phase is considered a safe option if suitable molecular monitoring is available. However, the question arises as to which factors can contribute to the maintenance of TFR, and immunologic surveillance of the remaining leukemic cells is believed to be one of them. Argentina Stop Trial is an open-label, single-arm, multicenter trial assessing TFR after tyrosine kinase inhibitors interruption, that after more than 4 years showed a successful TFR rate of 63%.

**Methods:**

In this context, we set up an immunological study by flow cytometry in order to analyze specific NK cell subsets from peripheral blood patient samples both at the time of discontinuation as well as during the subsequent months.

**Results:**

At the time of discontinuation, patients show a mature NK cell phenotype, probably associated to TKI treatment. However, 3 months after discontinuation, significant changes in several NK cell receptors occurred. Patients with a higher proportion of CD56dim NK and PD-1+ NK cells showed better chances of survival. More interestingly, non-relapsing patients also presented a subpopulation of NK cells with features associated with the expansion after cytomegalovirus infection (expression of CD57+NKG2C+), and higher proportion of NKp30 and NKp46 natural cytotoxicity receptors, which resulted in greater degranulation and associated with better survival (p<0.0001).

**Discussion:**

This NK cell subset could have a protective role in patients who do not relapse, thus further characterization could be useful for patients in sustained deep molecular response.

## 1 Introduction

During the last two decades, tyrosine kinase inhibitors (TKIs) have drastically improved the outcome of chronic myeloid leukemia (CML) patients in chronic-phase (CP) (1). Currently, in Argentina four TKIs have been approved for the treatment of CP-CML in frontline setting: imatinib (Glivec, Novartis), nilotinib (Tasigna, Novartis), dasatinib (Sprycel, Bristol-Myers Squibb) and bosutinib (Bosulif, Pfizer). Thanks to these agents, CML patients present survival similar to that of the general population (2). Treatment success is assessed by measuring the reduction of detectable *BCR-ABL1* mRNA levels in the peripheral blood (PB) to a point 4.0 or 4.5 logs below the International Scale baseline (3, 4), which is known as deep molecular response (DMR) (5). Accordingly, approximately half of CP-CML patients who achieve a durable DMR are able to sustain molecular remission after TKI withdrawal, making treatment-free remission (TFR) a therapeutic goal for these patients. A number of randomized controlled trials, as well as real-world studies, have shown that between 35–65% of patients successfully discontinue therapy and achieve a long-lasting TFR (6–8). Based on these findings, several guidelines of TFR recommendations have been published (2, 9, 10). Nevertheless, to date, no robust predictors of successful TKI discontinuation have been identified in TFR clinical trials (11). Argentine Stop Trial (AST) is to date the largest clinical trial of patients in Latin America who stopped across all types of TKIs. In this ongoing multicenter trial, we demonstrated the feasibility of TFR in our country and we described several novel potential biomarkers useful to improve patients’ selection for discontinuation (12).

Given that leukemic stem cells (LSCs) persist even in patients with a complete molecular response (13), it is necessary to consider the role of the immune system in controlling disease progression in those patients who can maintain TFR. Several authors demonstrated that the patients who were able to sustain molecular remission without treatment, were those with a significantly higher number of CD56^dim^ Natural Killer (NK) cells at the time of discontinuation (14, 15), suggesting that NK cells could play an important role in the control of LSCs after discontinuation of therapy. In recent years, there has been increasing interest in NK cell subpopulations believed to have adaptive or memory-like features in terms of greater functionality in the face of a second encounter or challenge. The first evidence of this type of cells is presented in the context of cytomegalovirus (HCMV) infection, where there is an expansion of a long-lived subset that displays a mature phenotype (CD56^dim^CD57^+^), and expresses high levels of the activation receptor NKG2C (16, 17). This subset is also characterized by the absence of expression of the intracellular adaptor protein FcϵRIγ, resulting in decreased expression of the natural cytotoxicity receptors (NCRs) NKp30 and NKp46 and consequently lower natural cytotoxicity (18, 19). Nevertheless, the expression of NKG2C does not exactly overlap with the loss of FcϵRIγ, thus there is certain heterogeneity within memory like NK cells (20).

In the context of hematological malignancies, Cichocki et al. described the presence of a CD56^dim^CD57^+^NKG2C^+^ subpopulation of NK cells that expanded in response to HCMV reactivation early after hematopoietic cell transplant. Interestingly, this was associated with reduced risk of relapse one year after transplant and improved disease free survival compared to HCMV^+^ recipients without reactivation or HCMV^-^ recipients. Additionally, this subpopulation exhibited higher TNFα and IFNγ production (21). Moreover, a substudy of the EURO-SKI discontinuation trial showed that, in imatinib-treated patients, the non-relapsing group presented higher frequencies of mature adaptive-like NK cells with enhanced cytokine secretion capacity (15).

Taking into account the role of the immune system, it is of great interest to characterize NK cells of patients who discontinue therapy in order to seek differences between those that can sustain a deep molecular response without therapy and those who suffer a molecular relapse. In this study, we present evidence suggesting that the presence of a subpopulation of mature NK cells with features associated to the expansion after HCMV infection and NCR expression might have a role in successful discontinuation of TKI treatment.

## 2 Materials and methods

### 2.1 Study design and patients’ baseline characteristics

The study was conducted as a sub-study of the Argentina Stop Trial (AST) registered in the National Registry of Health Research (RENIS: ISO02688) (12). For this prospective, single-arm, open-label, nonrandomized trial, we enrolled 46 patients with CP-CML at 7 Argentinean centers in Buenos Aires city and 3 more provinces (Buenos Aires province, Mendoza and Entre Rios). PB samples were collected once a month for the first 6 months after discontinuation of TKI, then every 2 months until month 12, and every 3 months thereafter for at least 2 years. Molecular response was assessed with RT-qPCR at 2 designated standardized laboratories (“Programme for harmonization to international scale”) (22, 23), and reported as the ratio of *BCR-ABL1* to *ABL1* on the International Scale. Molecular recurrence was defined as loss of major molecular response (MMR), corresponding to expression of more than 0.1% *BCR-ABL1* **^IS^** transcripts at any time. For patients with loss of MMR, treatment was restarted with the same TKI. PB samples from healthy donors (HD) (n=10) were included for comparative purposes. All patients and HD gave written informed consent.

Twenty-three patients (50%) were females and the Sokal score was low in 22 patients (47.8%). Patients were treated with imatinib (n = 37), dasatinib (n = 4) or nilotinib (n = 5) with branded (n = 35) or generic (n = 11) TKIs. Median duration of treatment was 128 months (range 52 - 244 months) and median duration of DMR (detectable BCR-ABL1IS ≤ 0.01%, or undetectable BCR-ABL1 with 10,000 or more ABL1 transcripts, i.e. at least MR4.0 or better) was 70 months (range 30 - 147 months).

### 2.2 Immunophenotyping of NK cells

**Figure 1:**
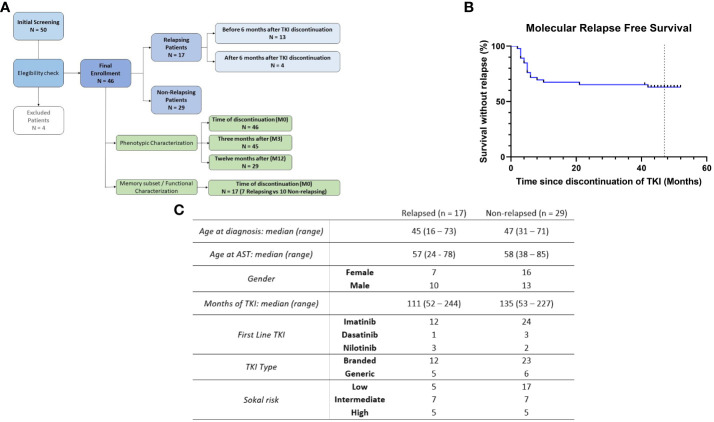
Organization of the study and characteristics of the patients. **(A)** Flowchart summarizing the distribution of patients according to timepoints and clinical status. **(B)** Molecular recurrence-free survival after TKI discontinuation (N = 46). **(C)** Patients baseline characteristics.

PB samples were obtained before stopping TKI and then at months 3, 12 and at any time when MMR was lost, as shown in **Figure 1A**. Peripheral blood mononuclear cells (PBMCs) were isolated by density-gradient centrifugation (Ficoll-Paque PLUS, GE Healthcare Life Sciences) for 30 minutes at 1,500g, followed by a washing step in physiological solution, and centrifugation at low speed (10 minutes at 1,300g). 2x10^6^ PBMCs were separated to determine NK cell subpopulations by flow cytometry, and the remaining cells were cryopreserved in heat-inactivated Fetal Bovine Serum (FBS) (Gibco, Thermo Fisher Scientific) plus 10% dimethyl sulfoxide (DMSO).

The following anti–human monoclonal antibodies (mAbs) were used in this part of the study: BV421 anti-CD56 (NCAM16.2), APC-H7 anti-CD3 (SK7), APC anti-IgG1 (X-40, MOPC-21), PE anti-IgG2b (MPC-11), PE anti-IgG2a (X-39), BB515 anti-NKp44 (p44-8), V500 anti-CD16 (3G8), PE-Cy7 anti-CD25 (M-A251), APC anti-NKG2D (1D11), FITC anti-CD57 (NK-1), APC anti-PD-1 (MIH4), PE anti-CD158a (HP-3E4), PE anti-CD158b (CH-L), PE anti-NKp30 (p30-15), PE-Cy7 anti-NKp46 (9-E2), all from BD Biosciences (San Jose, CA, USA); PE anti-NKG2A (131411; R&D Systems) and AF647 anti-NKG2C (134591; R&D Systems). PBMC were stained for 30 min at 4°C, washed twice with staining buffer (PBS with 2% FBS) and acquired in a FACSCanto II flow cytometer (BD). Flow cytometric data were analyzed using FlowJo software (Tree Star).

### 2.3 *In vitro* NK cell cytokine secretion and degranulation

PBMCs from patient samples at the time of discontinuation were thawed and incubated in RPMI 1640 medium supplemented with 10% heat-inactivated FBS (GIBCO, New Zealand). 2 mM L-glutamine and 100 U/mL penicillin/streptomycin (complete RPMI, all from Gibco, Carlsbad, CA, USA) plus 200U/ml DNase (Sigma Aldrich, St. Louis, Missouri, US) for 1 hour at 37°C in a humid atmosphere containing 5% CO_2_. Then PBMC were washed with PBS (Gibco, Thermo Fisher Scientific) and resuspended in complete RPMI medium. Cells were cultivated in complete RPMI Medium with 0.3 ng/ml of human rhIL-15 (PeproTech, New Jersey, US) overnight (ON) at 37°C in a humid atmosphere containing 5% CO_2_.

3×10^5^ PBMC were cultured with K562 cell line (CCL-243, ATCC) with complete RPMI medium. The number of target cells was calculated based on the percentage of NK cells, so the co-cultures were performed at a ratio of 1:5 NK:K562 cells. Cells were incubated for 6 h at 37°C in 5% CO2, with anti-CD107a (clone H4A3, BD) added at the beginning of the assay and Protein Transport Inhibitor (Golgi Stop, BD) added after the first hour. Cells were harvested, stained with the viability marker FVS 510 (BD) for 15 min at RT, washed and labeled with BV421 anti-CD56, APC-H7 anti-CD3, AF647 anti-NKG2C, PE-Cy7 anti-NKp46 and PerCP-Cy5.5 anti-HLA-DR for 15 min at RT. Cells were then fixed and permeabilized using Fixation and Permeabilization kit (BD) according to manufacturer’s protocol, and then labeled with PE anti-IFNγ (4S.B3, BD Biosciences) for 30 min at 4°C. Cell acquisition and analysis were performed as detailed above. Basal degranulation and IFNγ production were determined in the absence of target cells.

### 2.4 Measurement of IgG antibodies against human cytomegalovirus

Anti-HCMV IgG titer was determined quantitatively in plasma samples at the Alexander Fleming Institute Clinical Analysis Laboratory by the enzyme-linked fluorescence assay technique using the VIDAS CMV IgG kit (Biomerieux) according to the manufacturer’s instructions. Results were expressed in arbitrary units/mL (AU/mL) as negative (< 4.0 AU/mL) or positive (≥ 4.0 AU/mL) with no ambiguous range.

### 2.5 Statistical analyses

Analysis of molecular recurrence-free survival was performed using Graphpad Prism (GraphPad software, version 8.0.1). To compare variables between groups, the Mann Whitney test or the Wilcoxon test was performed when appropriate. Quantitative variables were dichotomized using the ROC curves, and cut-off points were established according to the highest likelihood ratio, maximizing specificity. Survival curves were compared using the log-rank test. Correlation was assessed with the Spearman test. P values < 0.05 were considered statistically significant.

## 3 Results

### 3.1 Phenotypic characterization of NK cells at the time of discontinuation and changes through time

Among the forty-six patients enrolled in this study, seventeen (37%) lost MMR (**Figure 1A**), resulting in a molecular relapse-free survival of 63% at 48 months (**Figure 1B**). For these patients, baseline characteristics and treatment information are shown in **Figure 1C**. Regarding clinical variables, only prior time in deep molecular response (DMR) and treatment duration were significantly associated with the probability of successful TFR in our cohort, as we have recently published (12). Since NK cell receptors play a key role in target recognition and transmission of activating or inhibitory signals after binding to their ligands, we thus decided to examine the expression of a large panel of NK cell receptors, both at the time of discontinuation as well as at specific subsequent time points (3 and 12 months).

We first compared NK cell proportions in AST patients and healthy donors (HD) (**Supplementary Table 1**). The median proportion of NK cells (CD3^-^CD56^+^) among lymphocytes was significantly increased in patients (p = 0.0043), together with CD16^+^ NK cells and CD57^+^ NK cells (p = 0.0046 and p = 0.0208, respectively), suggesting an immunomodulatory effect of TKIs, resulting in a more mature phenotype. On the contrary, PD-1^+^ NK cells were decreased in AST patients (p <0,0001), but with low percentages of expression (**Supplementary Figure 1**). There were no other statistically significant differences.

**Figure 2:**
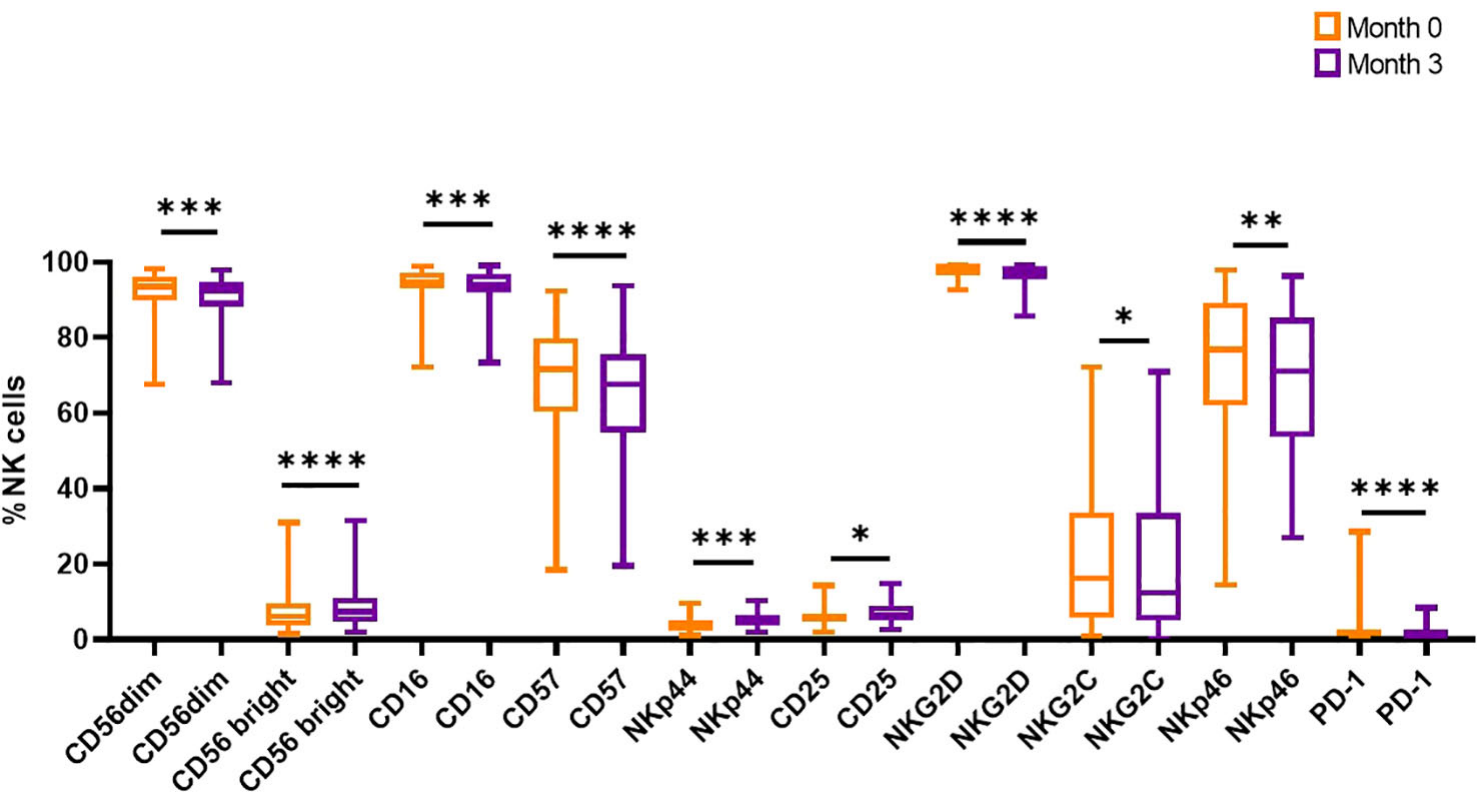
Comparison between NK cell markers from the time of discontinuation and three months after. Wilcoxon tests were performed (**** p<0.0001, *** p<0.001, ** p<0.01, *p<0.05).

To determine if the absence of the TKI could affect the expression of these markers, patient samples at the time of discontinuation were compared with samples obtained three months after treatment interruption and analyzed regardless of patient clinical status (**Supplementary Table 2**). It should be noted that one patient relapsed at month 2 therefore we do not have the sample corresponding to month 3, thus resulting in a total of 45 patients for this time point. Ten out of 14 receptors resulted significantly changed, suggesting a very dynamic behavior of this cell compartment, with significantly increased levels of CD56^bright^, NKp44 and CD25 but decreased levels of CD56^dim^, CD16, CD57, NKG2D, NKG2C, NKp46 and PD-1 (**Figure 2**).

**Figure 3:**
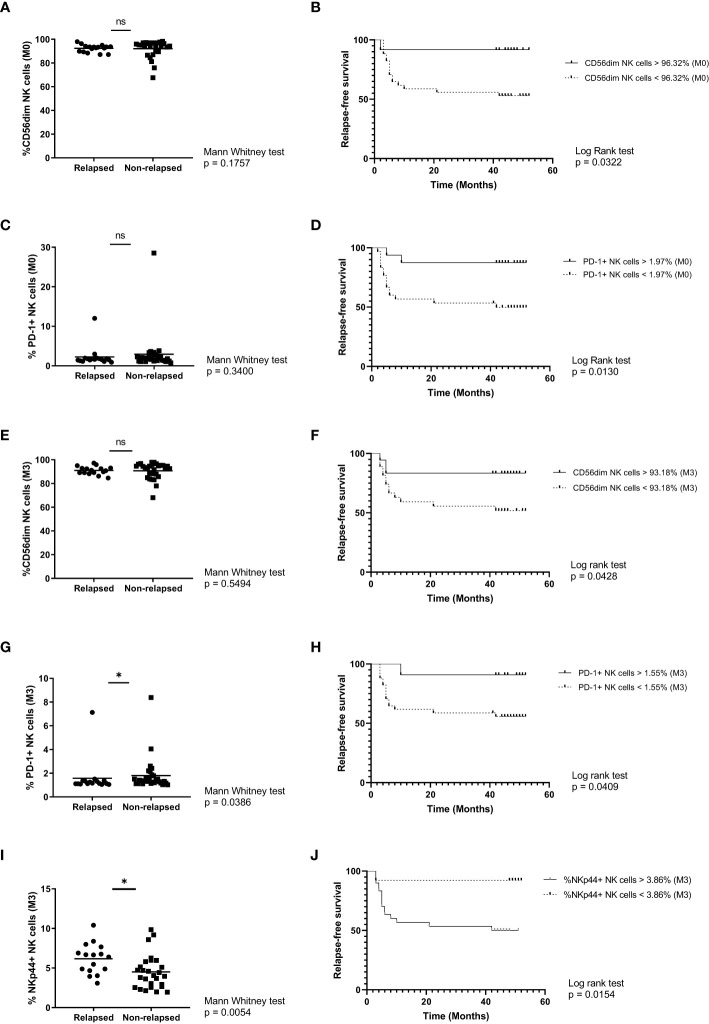
Phenotypic characterization of NK cell markers from relapsing and non-relapsing patients. **(A)** Percentage of CD56^dim^ NK cells in relapsed vs non-relapsed patients at the time of discontinuation. **(B)** Molecular recurrence-free survival according to percentage of CD3^-^CD56^dim^ NK cells at the time of discontinuation. **(C)** Percentage of PD-1^+^ NK cells in relapsed vs non-relapsed patients at the time of discontinuation. **(D)** Molecular recurrence-free survival according to percentage of PD-1^+^NK cells at the time of discontinuation. **(E)** Percentage of CD56^dim^ NK cells in relapsed vs non-relapsed patients at month 3 after stopping TKI. **(F)** Molecular recurrence-free survival according to percentage of CD3^-^CD56^dim^ NK cells after 3 months of TKI discontinuation. **(G)** Percentage of PD-1^+^ NK cells in relapsed vs non-relapsed patients at month 3 after stopping TKI. **(H)** Molecular recurrence-free survival according to percentage of PD-1^+^ NK cells after 3 months of TKI discontinuation. **(I)** Percentage of NKp44^+^ NK cells in relapsed vs non-relapsed patients at month 3 after stopping TKI. **(J)** Molecular recurrence-free survival according to percentage of NKp44^+^ NK cells after 3 months of TKI discontinuation. Mann-Whitney and Log-rank tests were performed (* p<0.05, ns: non-significant).

Next, our aim was to analyze the same panel of NK receptors in relapsing and non-relapsing patients at the time of discontinuation. By comparing the mean expression values between groups, no statistically significant differences were found for any of the activating receptors (CD16, NKG2C, NKG2D, NKp30, NKp44, NKp46), the inhibitory receptor NKG2A, CD158a/b from the Killer cell immunoglobulin-like receptor (KIR) family, the interleukin-2 receptor CD25 and the maturation marker CD57 (**Table 1**). Interestingly, although there were no statistically significant differences regarding the percentage of CD56^dim^ and PD-1^+^ NK cells between groups (**Figures 3A, C**), here we report that molecular relapse free survival (RFS) time was significantly longer for those patients showing higher frequencies of these subsets (Log-rank (Mantel-Cox) Test p=0.0322 and p=0.0130, respectively) (**Figures 3B, D**).

**Table 1:** Percentage of NK cells and NK cell receptors at the time of discontinuation among relapsing and non-relapsing patients.

	*Relapsed (n = 17)*		*Non-Relapsed (n = 29)*		*R vs NR (p-value)*
	Median	Range	Median	Range	
*NK cells*	16.27	8.75 - 35.15	14.42	4.61 - 44.30	0.6163
*CD56^dim^*	93.31	87.13 - 97.96	94.38	67.52 - 98.31	0.1757
*CD56^bright^*	6.26	2.02 - 12.19	5.16	1.36 - 30.95	0.1417
*CD16*	94.75	89.25 - 99.30	96.55	72.10 - 98.95	0.6241
*CD57*	70.625	18.43 - 85.15	72.2	31.48 - 92.38	0.4534
*NKp44*	4.2	1.99 - 9.65	3.09	1.13 - 9.38	0.4952
*CD25*	6.09	2.89 - 14.3	5.35	1.97 - 13	0.5466
*NKG2A*	10.5	2.22 - 74.50	9.63	2.73 - 93.00	0.8088
*NKG2D*	98.9	92.5 - 99.7	98.6	93.00 - 99.70	0.4258
*NKG2C*	11.3	0.715 - 89.30	20.75	0.97 - 72.15	0.3911
*CD158a/b*	34.3	26.4 - 74.70	48.3	15.70 - 72.8	0.4330
*NKp30*	79.1	31.7 - 98.5	65.6	13.6 - 96.70	0.3035
*NKp46*	76.8	32.90 - 97.9	75.4	14.40 - 95.90	0.7143
*PD-1*	1.65	0.92 - 12	1.89	0.77- 28.5	0.3400

NR (non-relapsed); R (relapsed); Median concentrations and range in percentage are listed. P-values were derived from Mann-Whitney test.

To assess how durable the immune profile could be, these comparisons were repeated with samples at 3 months after TKI cessation. Again, there were no statistically significant differences regarding the percentage of CD56dim (**Figure 3E**). Both NK subsets (CD56^dim^ and PD-1^+^) maintain significance in terms of RFS (**Figures 3F, H**) and in addition for the PD-1^+^ subset statistically significant difference was reported between mean groups (**Figure 3G**). Finally, at this time point analyses showed significant alteration only in the expression of NKp44, after TKI discontinuation. Both Mann Whitney (**Figure 3I**) and Log rank tests (**Figure 3J**) showed a significant difference at 3 months after discontinuation between relapsing and non-relapsing patients.

Considering that TKIs exert off-target effects on NK cells, and given that we have observed certain differences in the expression of several markers between the time of discontinuation and three months after, we thus wondered if the absence of the inhibitor affected relapsed and non-relapsed patients differently and if those differences could help to identify future relapses. Remarkably, several markers were down or upregulated between the time of discontinuation and the first three months off-TKI (**Table 2**), but only CD57, NKp44, NKG2D and PD-1 showed the same tendency and significance for both groups (**Supplementary Figure 2A**). To address whether this change was maintained over time, immunophenotyping was also repeated 12 months after TKI cessation for the non-relapsing group (**Supplementary Table 3**). Interestingly, those changes remained after one year without treatment (**Supplementary Figure 2B**) but also changes in NKp46 and NKG2C expression were observed.

**Table 2:** Percentage of NK cells and NK cell receptors in relapsed and non-relapsed patients at the time of discontinuation and three months after.

	*Relapsed (n = 16)*					*Non-Relapsed (n = 29)*				
	*Median M0*	*Range M0*	*Median M3*	*Range M3*	*M0 vs M3 (p-value)*	*Median M0*	*Range M0*	*Median M3*	*Range M3*	*M0 vs M3 (p-value)*
*NK cells*	16.175	8.75 - 31.93	15.43	7.65 - 51.72	0.9901	14.42	4.61 - 44.30	12.12	4.31 - 35.68	**0.0262**
*CD56^dim^*	93.07	87.13 - 96.29	90.995	84.61 - 97.22	0.0782	94.38	67.52 - 98.31	93.32	68.04 - 97.85	**0.0032**
***CD56^bright^***	6.515	3.42 - 12.19	8.765	2.7 - 14.09	**0.0425**	5.16	1.36 - 30.95	6.41	1.99 - 31.49	**0.0007**
*CD16*	94.725	89.25 - 97.40	93.6	90.25 - 98.05	0.2916	96.55	72.10 - 98.95	95.35	73.4 - 99	**0.0012**
***CD57***	68.115	18.43 - 85.15	62.065	19.48 - 81.95	**0.0008**	72.2	31.48 - 92.38	70.18	27.65 - 93.7	**0.0023**
***NKp44***	4.3	1.99 - 9.65	6.525	3.08 - 10.4	**0.0034**	3.04	1.13 - 9.38	4.06	1.94 - 9.84	**0.0162**
*CD25*	6.155	2.89 - 14.3	6.975	4.06 - 14.7	0.4332	5.35	1.97 - 13	6.5	2.65 - 13.8	**0.019**
*NKG2A*	11.2	2.22 - 74.50	13.05	2.92 - 80.1	0.3755	9.63	2.73 - 93.00	8.5	2.46 - 60.2	0.4581
***NKG2D***	98.85	92.5 - 99.7	98.4	85.7 - 99.4	**0.0245**	98.6	93.00 - 99.70	97.5	86.7 - 99.3	**0.001**
*NKG2C*	10.385	0.715 - 67.75	10.865	0.76 - 63.4	**0.0131**	20.75	0.97 - 72.15	20.35	0.23 - 70.95	0.2545
*CD158a/b*	34.2	26.4 - 67.60	33.4	22.4 - 64.7	**0.0428**	48.3	15.70 - 72.8	43.1	20.3 - 82.5	0.4582
*NKp30*	79.05	31.7 - 98.5	79.8	33.2 - 98.2	0.1365	66.3	13.6 - 96.70	64.45	22.2 - 97.4	0.8376
*NKp46*	77.55	32.90 - 97.9	64.4	35.4 - 96.3	**0.0056**	75.4	14.40 - 95.90	73.1	27 - 94.8	0.2916
***PD-1***	1.715	0.92 - 12	1.15	1.05 - 7.13	**0.0009**	1.89	0.77- 28.5	1.37	1.02 - 8.38	**0.0109**

M0 (month 0, time of discontinuation); M3 (month 3 after discontinuation); Median concentrations and range in percentage are listed. P-values were derived from Wilcoxon test. Bold values correspond to statistically significant results (p < 0.05) and receptors that share the same behavior between relapsed and non-relapsed patients.

### 3.2 NK cell subsets analysis reveals a subpopulation associated with better survival

**Figure 4:**
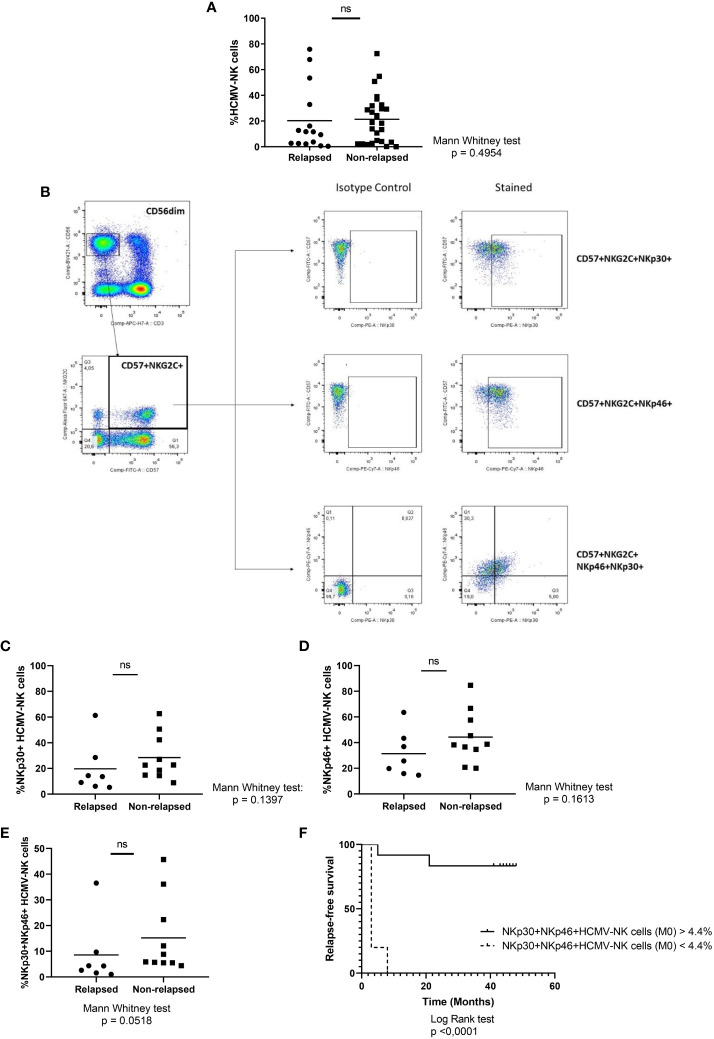
NK cell subsets analysis in relapsed vs non-relapsed patients at the time of discontinuation. **(A)** Percentage of CD56^dim^NKG2C^+^CD57^+^ (HCMV-NK cells). **(B)** Representative gating strategy of a patient with CD56^dim^NKG2C^+^CD57^+^ NK cell subset higher than 5%. **(C)** Percentage of CD56^dim^CD57^+^NKG2C^+^NKp30^+^ NK cells (NKp30^+^HCMV-NK cells). **(D)** Percentage of CD56^dim^CD57^+^NKG2C^+^NKp46^+^ NK cells (NKp46^+^HCMV-NK cells). **(E)** Percentage of CD56^dim^CD57^+^NKG2C^+^NKp30^+^NKp46^+^ NK cells (NKp30^+^NKp46^+^HCMV-NK cells). **(F)** Molecular recurrence-free survival according to percentage of NKp30^+^NKp46^+^HCMV-NK cells. Mann-Whitney test and Log-rank test were performed when appropriate. P-values lower than 0.05 were considered statically significant. ns, non-significant.

After human cytomegalovirus (HCMV) infection, there is an expansion of a certain NK cell subset characterized by the expression of CD57 and NKG2C among CD56^dim^ NK cells (HCMV-NK) (16, 17, 21). To determine the presence of this subpopulation, HCMV status was assessed in plasma samples of TFR patients, resulting in 42 seropositive (15 relapsed and 27 non-relapsed) and 4 seronegative (2 relapsed and 2 non-relapsed) patients. Taking into account only seropositive patients, there were no statistically significant differences among HCMV-NK cells between groups (**Figure 4A**). A great dispersion of data was observed, thus to deepen the characterization of this subset, we evaluated the coexpression of two NCRs according to our cytometry panel design, NKp30 and NKp46. In order to have more reproducible results, only seropositive patients whose HCMV-NK cell compartment was 5% or higher were considered for this analysis. This is because in those patients with HCMV-NK cells greater than 5%, the analysis is more robust, as can be seen in the gating strategy from **Figure 4B**, compared to the ones with less than 5%, with fewer events to analyze (**Supplementary Figure 3**). Consequently, only 17 patients met the inclusion criteria for this analysis, 7 in the relapsing group and 10 in the non-relapsing group. Although there is a tendency of higher frequencies of CD56^dim^NKG2C^+^CD57^+^NKp30^+^ (NKp30+ HCMV-NK) cells and CD56^dim^NKG2C^+^CD57^+^NKp46^+^ (NKp46+ HCMV-NK) cells in non-relapsing patients, no significant differences could be reported (**Figures 4C, D**, respectively). Remarkably, the simultaneous coexpression of NKp30 and NKp46 on CD56^dim^CD57^+^NKG2C^+^ subpopulation (NKp30+NKp46+HCMV-NK cells) was increased in non-relapsing patients (**Figure 4E**), and this subset was significantly associated with longer RFS (**Figure 4F**). At the 3- month and 12-months’ time points after TKI discontinuation there are no statistically significant changes over time (data not shown), suggesting that these subsets are not affected by the absence of TKI.

### 3.3 Degranulation of NKp30^+^NKp46^+^HCMV-NK cells is associated with improved survival

**Figure 5:**
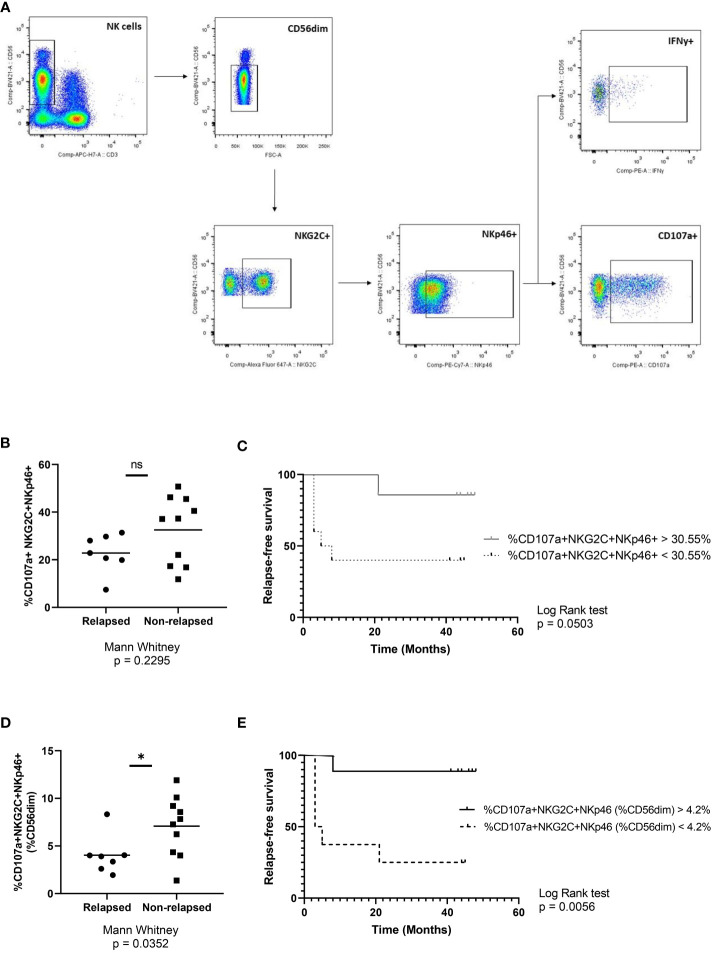
Functional role of CD56^dim^NKG2C^+^NKp46^+^ NK cells. **(A)** Representative gating strategy used for degranulation (CD107a) and IFNγ production assays. **(B)** Percentage of CD107a^+^ cells in the NKG2C^+^NKp46^+^ NK subpopulation at the time of discontinuation in relapsing and non-relapsing patients. **(C)** Molecular recurrence-free survival according to percentage of CD107a^+^ cells in the NKG2C^+^NKp46^+^ NK subpopulation. **(D)** Percentage of NKG2C^+^NKp46^+^CD107a^+^ cells as a proportion of CD56^dim^ NK cells at the time of discontinuation in relapsing and non-relapsing patients. **(E)** Molecular recurrence-free survival according to percentage of NKG2C^+^NKp46^+^CD107a^+^ cells as a proportion of CD56^dim^ NK cells. Mann-Whitney and Log-rank tests were performed (* p<0.05; ns, non-significant).

Finally, to better understand the relevance of this subpopulation, functional assays were performed using K562 cell line as target and analysis was conducted as shown in **Figure 5A**. No statistically significant differences were found between response groups in terms of degranulation or IFNγ production in NK cell population (data not shown). Taking into account that NKp30 and NKp46, show a very strong correlation (**Supplementary Figure 4**) and that unfortunately we could not use all the original markers in the functional assays due to panel design and technical limitations of the cytometer, we sought to determine the functionality of CD56^dim^NKG2C^+^NKp46^+^ NK cells, considering that this subset is analogous to that defined before. There were no differences between groups when analyzing the percentage of CD107a^+^ among this subpopulation (**Figures 5B, C**). However, when comparing the NKG2C^+^NKp46^+^CD107a^+^ NK cells as a percentage of CD56^dim^, the non-relapsing group presented higher levels of degranulation (**Figure 5D**) and also showed better chances of survival (**Figure 5E**) (Log-rank (Mantel-Cox) Test p=0.0056). Finally, IFNγ production showed no statistically significant differences between groups (**Supplementary Figure 5**).

## 4 Discussion

The mechanisms behind successful TFR are still unclear. Here, we were able to investigate systematically the frequencies of specific NK cell subpopulations, in sustained remission after treatment cessation. In our study, increased molecular relapse-free survival was associated with higher percentages of CD56^dim^ NK cells, and particularly PD-1^+^ NK cells both at the time of discontinuation and also at 3 months after stopping treatment. It has been reported that NK cells from patients with multiple myeloma or patients with post transplantation lymphoproliferative disorders may present low PD-1 levels (24), but expression of PD-1 by NK cells from CML patients has been poorly defined. Several studies suggest that PD-1 expression is increased on activated NK cells with a “more responsive phenotype” (25), which could explain why the non-relapsing patients express higher levels of this receptor. Moreover, the expansion of NKG2C^+^ NK cells was found to be associated with increased expression of the classical exhaustion markers LAG-3 and PD-1 upon chronic activation (26). Nevertheless, this was not the case for our cohort.

None of the remaining markers were differentially expressed between relapsed and non-relapsed patients, which could indicate that patients included in our study present NK cells with a competent phenotype. Nevertheless, thanks to having consecutive samples during the trial, we were able to evaluate the dynamics of their expression over time. The only receptor that showed differences between groups was NKp44 at month 3, a NCR only present on activated or cytokine stimulated NK cells, in contrast to NKp30 and NKp46 that are constitutively expressed on resting NK cells. Our results indicate higher expression of NKp44 in the relapsed group and better relapse-free survival in patients with lower expression of this marker. The role of this receptor in cancer is poorly understood, but it is well known that it can be expressed in three transcriptional variants that differ in the length of the cytoplasmic domain. The NKp44-1 isoform containing an ITIM motif has the potential for both inhibitory and activating signalling (27). The local cytokine profile could influence NKp44 splicing and together with the expression of certain ligands in target cells, determine the associated functional characteristics (28). Taking this into account, we could speculate that the clinical outcome of our cohort could be partially linked to the isoform expressed, which is in line with findings from Shemesh and col, where NKp44-1 variant expression has been related with poor survival in AML patients (29). Furthermore, when comparing the time of discontinuation and three and/or twelve months later, NKp44 along with three other receptors (CD57, NKG2D and PD-1) were found to share the same behaviour in relapsed and non-relapsed patients, probably due to the TKI withdrawal. In fact, TKIs not only directly inhibit BCR-ABL1 tyrosine kinase, but also exhibit immunomodulatory effects. Several reports show that the TKIs off-target effects range from clonal lymphocytosis (30) to fast NK and T cell mobilization in blood together with a more effective transmigration and enhanced cytotoxicity, and a consequent better clinical outcome (31). Though it is worth mentioning that the majority of these effects were seen in dasatinib-treated patients while our cohort was mostly treated with imatinib (80% of patients). On the other hand, this could also be linked to the fact that we and others (15) have found a higher percentage of NK cells in these patients compared to healthy donors. In our cohort, we have also found higher proportions of CD16^+^ NK cells and CD57^+^ NK cells, suggesting an immunomodulatory effect of these agents, to a more mature profile, although there is no previous evidence of modulation of specific markers that we know of. Unfortunately, in the AST study, no provision was made for obtaining blood counts on the day each sample was taken, so it was not feasible to determine these percentages in terms of absolute values of cells per cubic mm. Next, our aim was to investigate the presence of specific NK cell subsets by combining the expression of several markers, with particular interest in a subpopulation that is believed to expand after HCMV infection: CD56^dim^CD57^+^NKG2C^+^ NK cells (HCMV-NK). In our cohort, even though HCMV-NK cells did not differ between groups (**Figure 5A**), we noted that non-relapsing patients showed an increased expression of NKp30 and NKp46 within this subset, which also associated with better relapse-free survival. Functional assays by measuring degranulation (%CD107a) and IFNγ production in NK cells were performed but similarly to what previously reported Rea et al. (14), no significant differences were found between relapsing and non-relapsing patients, which corresponds to competent NK cells. In particular, the degranulation of the CD56^dim^NKG2C^+^NKp46^+^ subset was similar in both groups. Interestingly, when these events were reported as a percentage of CD56^dim^ NK cells, non-relapsed patients presented a higher proportion of the CD107a^+^NKG2C^+^NKp46^+^ compartment, and a significantly longer time of RFS. This could be explained by considering that a greater proportion of CD56^dim^ is present in molecular relapse-free patients and eventually a higher proportion of CD56^dim^NKG2C^+^NKp46^+^ NK cells contribute to the elimination of the residual leukemic cells. Unfortunately, the residual LSCs are very difficult to detect as well as the characterization of their surface markers that function as ligands for the effector NK cells; this could explain why, despite some patients have abundant NKp30^+^NKp46^+^HCMV-NK cells, they were unable to sustain MR over time. In this approach, another limitation may come from the use of the K562 cell line as target, which although it corresponds to CML, it was obtained from a patient in blast crisis, which represents a different clinical scenario. However, the K562 cell line is widely considered a gold standard for the evaluation of NK cell functionality, not only in oncohematology, but also in solid tumour immunology, because of its MHC class I deficiency (32). Another possible reason for not being able to visualize stronger differences could be the small number of patients that we were able to use for these analyses. That is why it would be of great interest to expand the cohort to confirm or reject these observations.

To our knowledge, our study is the first to describe this subset of NK cells with overall increased degranulation capacity, which appears to confer certain protective capacity for patients who do not relapse. However, the clinical impact of NK cells in patients who discontinued TKIs remains controversial, and further studies in a larger cohort are still needed. Finally, our results suggest that enhancing or restoring NK immune effector functions may be a strategy to increase the rate of successful TFR in CML.

## Data availability statement

The raw data supporting the conclusions of this article will be made available by the authors, without undue reservation.

## Ethics statement

The studies involving humans were approved by Comité de Ética del Instituto Alexander Fleming. The studies were conducted in accordance with the local legislation and institutional requirements. The participants provided their written informed consent to participate in this study.

## Author contributions

MB developed the study, conceived and planned the experiments, design and wrote the article. CP, BM, and AV conceived and developed the study. MS and BV processed patient samples, performed the experiments and statistical analysis of data and contributed to the writing and revision of all drafts. IG and MVer did the PCR analyses in the standardized national laboratories. EL helped designing flow cytometry panels, analyzing flow cytometry data, interpreted results and provided feedback on the manuscript. JM and JS participated in the analysis and interpretation of the data, contributed to the writing and revision of all drafts. MJ, RC, IF, MF, MVen, GB, RM, MO, MP, and CF led recruitment, treatment, and data collection in their study centers. All authors contributed to the article and approved the submitted version.
