# Supplementary material for: In-depth characterization of NK cell markers from CML patients who discontinued tyrosine kinase inhibitor therapy
Source: Front Immunol. 2023 Sep 25;14:1241600. doi: 10.3389/fimmu.2023.1241600 (PMC10561287; doi:10.3389/fimmu.2023.1241600)
Supplement: Supplementary file 1 [file DataSheet_1.docx]

Supplementary Material

# Supplementary Tables

**Supplementary Table 1: Percentage of NK cells and NK cell receptors in healthy donors and AST patients at the time of discontinuation**

|  | *Healthy donors (n = 10)* | | *AST patients (n = 46)* | | *Mann-Whitney (p-value)* |
| --- | --- | --- | --- | --- | --- |
|  | *Median* | *Range* | *Median* | *Range* |  |
| *NK cells* | 8.375 | 3.97 - 16.83 | 14.83 | 4.61 - 44.3 | **0.0043** |
| *CD56^dim^* | 92.55 | 61.5 - 96.3 | 93.57 | 67.52 - 98.31 | 0.1064 |
| *CD56^bright^* | 7.3 | 3.4 - 38.5 | 5.94 | 1.36 - 30.95 | 0.1438 |
| *CD16* | 90.675 | 65.95 - 96.75 | 94.975 | 72.10 - 99.3 | **0.0046** |
| *CD57* | 60.24 | 13.02 - 78.2 | 71.76 | 18.42 - 92.37 | **0.0208** |
| *NKp44* | 5.42 | 0.45 - 7.72 | 3.36 | 1.13 - 9.65 | 0.0924 |
| *CD25* | 5.765 | 3.58 - 14.9 | 5.63 | 1.97 - 14.3 | 0.9375 |
| *NKG2A* | 11.85 | 2.26 - 41.6 | 9.965 | 2.22 - 93 | 0.5740 |
| *NKG2D* | 98.35 | 81.3 - 99.5 | 98.8 | 92.5 - 99.7 | 0.2158 |
| *NKG2C* | 15.25 | 5.755 - 61.25 | 16.25 | 0.715 - 89.30 | 0.7627 |
| *CD158a/b* | 33.8 | 14.4 - 48.1 | 38.35 | 15.70 - 74.7 | 0.0677 |
| *NKp30* | 91.7 | 45.4 - 97.2 | 72.05 | 13.60 - 98.5 | 0.1123 |
| *NKp46* | 83.35 | 37.1 - 96.3 | 76.35 | 14.40 - 97.9 | 0.5168 |
| *PD-1* | 1.99 | 1.19 - 3.11 | 1.81 | 0.77 - 28.5 | **<0,0001** |

**Supplementary Table 2: Percentage of NK cells and NK cell receptors in AST patients at the time of discontinuation and three months after**

|  | *AST (n = 45)* | | | | |
| --- | --- | --- | --- | --- | --- |
|  | *Median M0* | *Range M0* | *Median M3* | *Range M3* | *M0 vs M3 (p-value)* |
| *NK cells* | 14.7 | 4.61 - 44.3 | 12.42 | 4.31 - 51.72 | 0.0900 |
| *CD56^dim^* | 93.56 | 67.52 - 98.31 | 92.47 | 68.04 - 97.85 | **0.0007** |
| *CD56^bright^* | 6.07 | 1.36 - 30.95 | 7.28 | 1.99 - 31.49 | **<0.0001** |
| *CD16* | 94.8 | 72.1 - 98.95 | 94.15 | 73.4 - 99 | **0.0010** |
| *CD57* | 71.63 | 18.43 - 92.38 | 67.58 | 19.48 - 93.7 | **<0.0001** |
| *NKp44* | 3.22 | 1.13 - 9.65 | 4.73 | 1.94 - 10.4 | **0.0001** |
| *CD25* | 5.77 | 1.97 - 14.3 | 6.56 | 2.65 - 14.7 | **0.0189** |
| *NKG2A* | 10.3 | 2.22 - 93 | 8.92 | 2.46 - 80.1 | 0.9488 |
| *NKG2D* | 98.8 | 92.5 - 99.7 | 97.7 | 85.7 - 99.4 | **<0.0001** |
| *NKG2C* | 16.1 | 0.71 - 72.15 | 12.4 | 0.23 - 70.95 | **0.0164** |
| *CD158a/b* | 37.2 | 15.7 - 72.8 | 37.5 | 20.3 - 82.5 | 0.1071 |
| *NKp30* | 72.05 | 13.6 - 98.5 | 73.3 | 22.2 - 98.2 | 0.1697 |
| *NKp46* | 76.8 | 14.4 - 97.9 | 71.1 | 27 - 96.3 | **0.0097** |
| *PD-1* | 1.81 | 0.77 - 28.5 | 1.31 | 1.02 - 8.38 | **<0.0001** |

**Supplementary Table 3: Percentage of NK cells and NK cell receptors in non-relapsing patients at the time of discontinuation and twelve months after**

|  | *Non-Relapsed (n = 29)* | | | | |
| --- | --- | --- | --- | --- | --- |
|  | *Median M0* | *Range M0* | *Median M12* | *Range M12* | *M0 vs M12 (p-value)* |
| *NK cells* | 14.42 | 4.61 - 44.30 | 15.22 | 4.71 - 36.7 | 0.4812 |
| *CD56^dim^* | 94.38 | 67.52 - 98.31 | 94.47 | 73.65 - 98.26 | 0.8314 |
| *CD56^bright^* | 5.16 | 1.36 - 30.95 | 5.25 | 1.56 - 26.08 | 0.8314 |
| *CD16* | 96.55 | 72.10 - 98.95 | 95.6 | 77.25 - 98.9 | 0.4387 |
| *CD57* | 72.2 | 31.48 - 92.38 | 66.5 | 27.1 - 92.78 | **<0,0001** |
| *NKp44* | 3.5 | 1.13 - 9.38 | 6.99 | 3.61 - 11.9 | **<0,0001** |
| *CD25* | 5.28 | 1.97 - 13 | 5.625 | 3.00 - 28.5 | 0.3608 |
| *NKG2A* | 9.63 | 2.73 - 93.00 | 12.1 | 5.04 - 60.5 | 0.0963 |
| *NKG2D* | 98.35 | 93.00 - 99.70 | 95.7 | 74.4 - 99.2 | **<0,0001** |
| *NKG2C* | 20.75 | 0.97 - 72.15 | 20.5 | 0.4 - 55.75 | **0.0008** |
| *CD158a/b* | 48.3 | 15.70 - 72.8 | 44.8 | 17.3 - 75 | 0.9384 |
| *NKp30* | 66.3 | 13.6 - 96.70 | 66.75 | 35.2 - 98.7 | 0.1998 |
| *NKp46* | 75.4 | 14.40 - 95.90 | 72.6 | 17.9 - 94.2 | **0.014** |
| *PD-1* | 1.89 | 0.77 - 28.5 | 1.3 | 1.00 - 12.9 | **0.0055** |

# Supplementary Figures

**
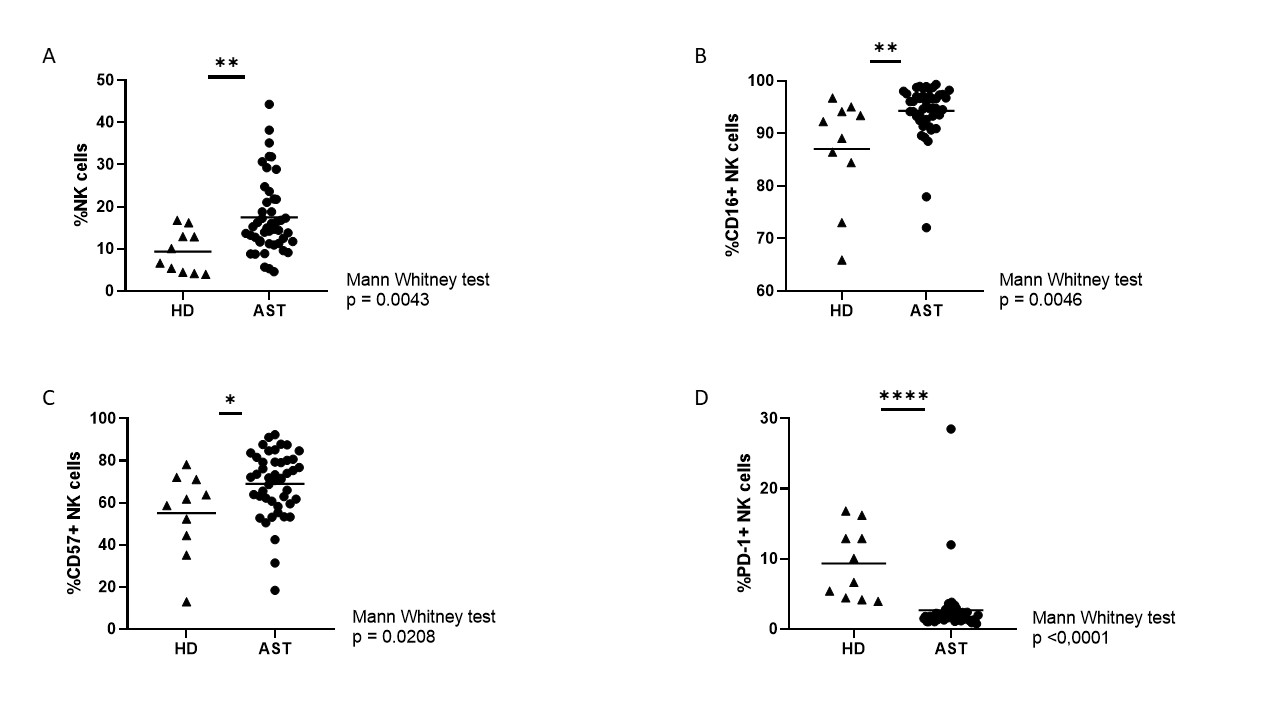
**

**Supplementary Figure 1.** **Comparison between healthy donors (HD) and AST patients (AST) at the time of discontinuation**. Percentage of CD3^-^CD56^+^ NK cells (**A**), CD16^+^NK cells (**B**), CD57^+^ NK cells (**C**) and PD-1^+^ NK cells (**D**). Mann-Whitney tests were performed (**** p<0.0001, *** p<0.001, ** p<0.01, *p<0.05).

**
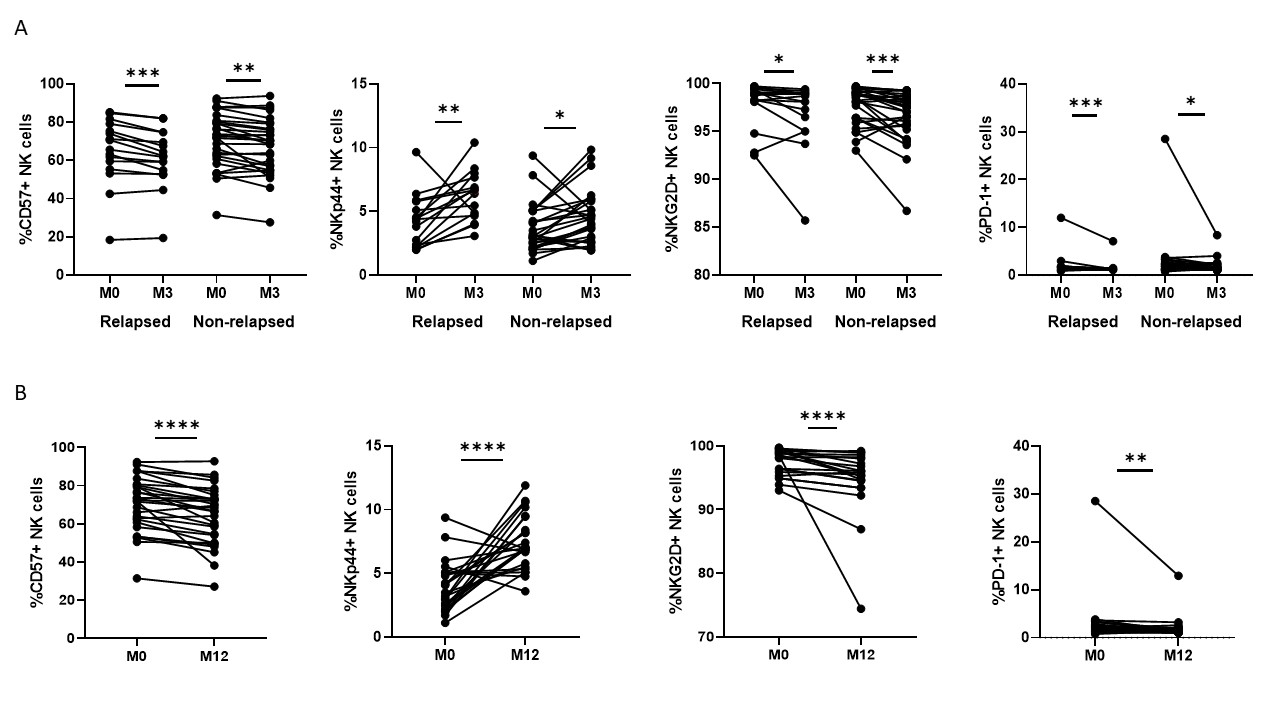
Supplementary Figure 2. Changes in receptors from the moment of the discontinuation to 3 and 12 months after. (A)** Percentage of CD57^+^, NKp44^+^, NKG2D^+^ and PD-1^+^ NK cells respectively, at discontinuation (M0) and three months later (M3), in relapsed and non-relapsed patients. **(B)** Percentage of CD57^+^, NKp44^+^, NKG2D^+^ and PD-1^+^ NK cells respectively, at discontinuation (M0) and twelve months later (M12), in non-relapsed patients. Wilcoxon tests were performed (**** p<0.0001, *** p<0.001, ** p<0.01, *p<0.05).


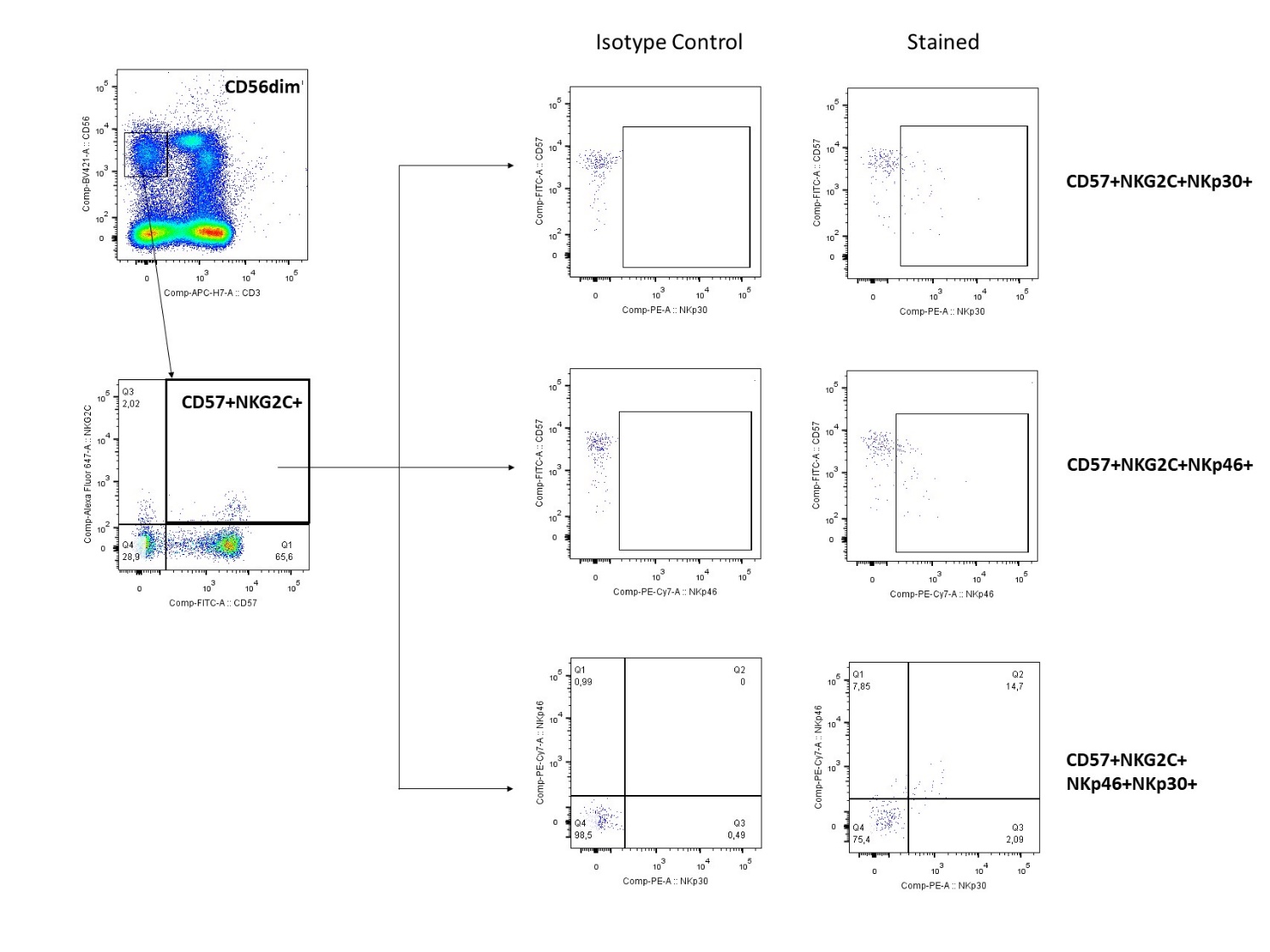
**Supplementary Figure 3.** Representative gating strategy of a patient with CD56^dim^NKG2C^+^CD57^+^ NK cell subset lower than 5%.


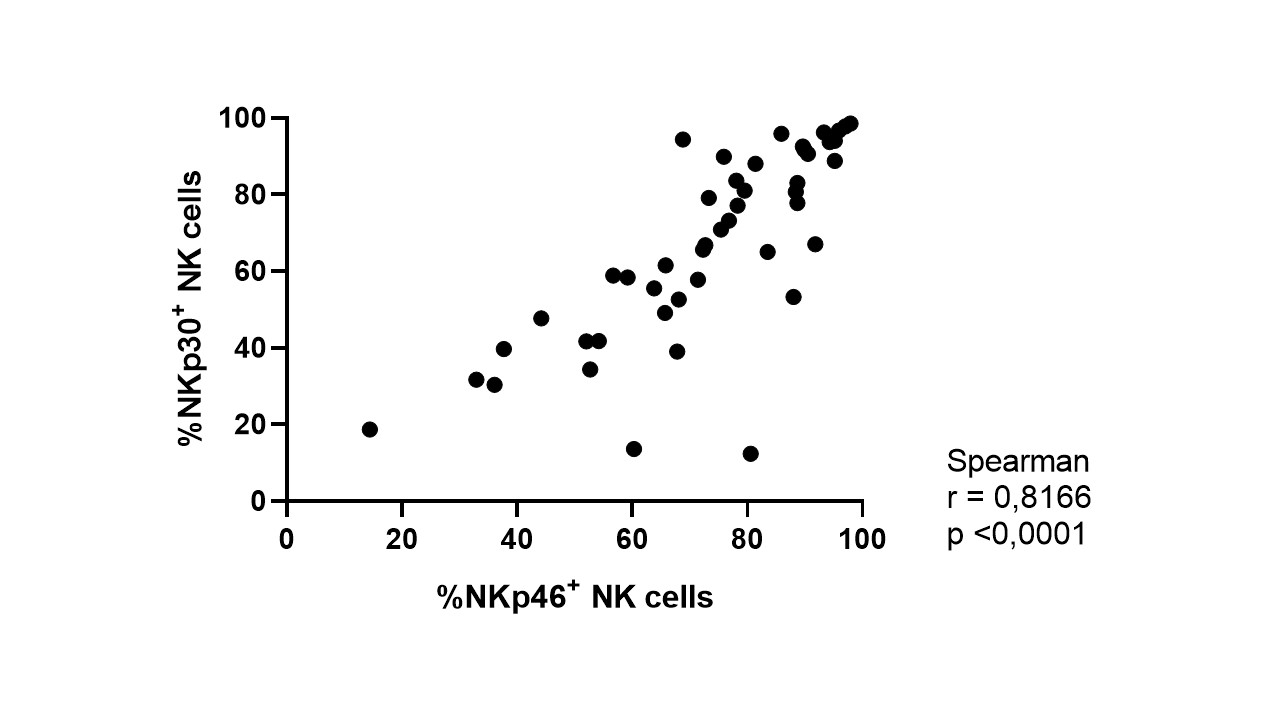
**Supplementary Figure 4.** Correlation analysis between NKp30 and NKp46 in NK cells at the time of discontinuation.


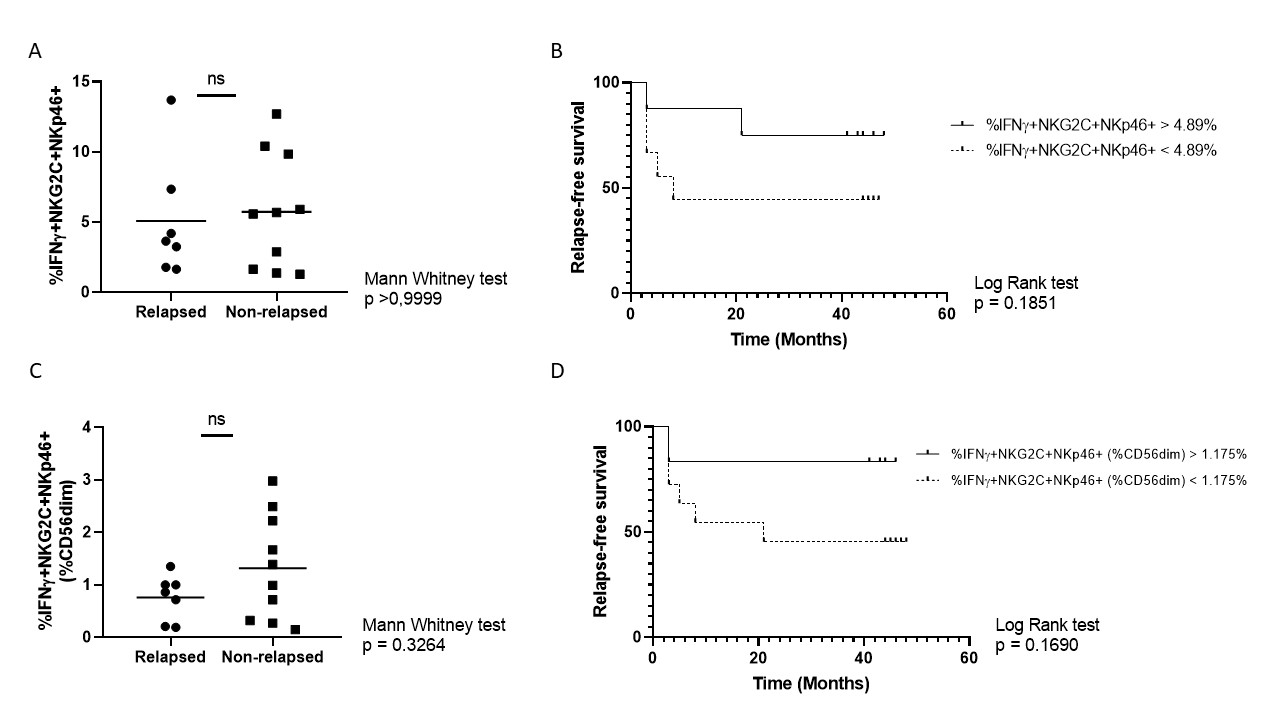


**Supplementary Figure 5. Memory NK cells and their functional role. (A)** Percentage of IFNγ^+^ cells in the NKG2C^+^NKp46^+^ NK subpopulation at the time of discontinuation in relapsing and non-relapsing patients. **(B)** Molecular recurrence-free survival according to percentage of IFNγ^+^ cells in the NKG2C^+^NKp46^+^ NK subpopulation. **(C)** Percentage of NKG2C^+^NKp46^+^IFNγ^+^ cells as a proportion of CD56^dim^ NK cells at the time of discontinuation in relapsing and non-relapsing patients. **(D)** Molecular recurrence-free survival according to percentage of NKG2C^+^NKp46^+^IFNγ^+^ cells as a proportion of CD56^dim^ NK cells. Mann-Whitney and Log-rank tests were performed (**** p<0.0001, *** p<0.001, ** p<0.01, *p<0.05).
